# Knowledge, attitudes and practices of university students toward COVID-19 in Southern region, Afghanistan: a cross-sectional study

**DOI:** 10.1186/s12909-023-04164-w

**Published:** 2023-03-20

**Authors:** Rahmatullah Sirat, Mohammad Sediq Sahrai, Bilal Ahmad Rahimi, Abdullah Asady, Abdul Wahed Wasiq

**Affiliations:** 1 Department of Microbiology, Faculty of Medicine, Kandahar University, Kandahar, 03801 Afghanistan; 2 Department of Internal Medicine, Faculty of Medicine, Kandahar University, Kandahar, Afghanistan; 3 Department of Pediatrics, Faculty of Medicine, Kandahar University, Kandahar, Afghanistan; 4 Department of Microbiology, Kabul University of Medical Sciences, Kabul, Afghanistan

**Keywords:** COVID-19, University students, Knowledge, Attitudes, Practices, Afghanistan, Second wave

## Abstract

**Background:**

Successful implementation of preventive measures and level of awareness in particular among university students in Afghanistan could play a crucial role in spreading the information for better control of the ongoing pandemic. Therefore, the aim of this study was to determine the level of knowledge, attitudes and practices (KAP) regarding COVID-19 among Kandahar university students.

**Methods:**

A cross-sectional study conducted among undergraduate students to investigate their KAP regarding COVID-19 from January to March, 2021. The data were collected using a structured questionnaire (socio-demographic characteristics, KAP questionnaire) by phone call interviews.

**Results:**

From 359 participants, 88.9% were males, 81.3% were single, 65.1% lived in urban areas, and more than 64% of the students were less than 23 years old. Overall, 86.6% of the study participants had adequate level of knowledge and 87.5% had positive attitudes toward COVID-19. Almost one-third of the participants had good practices regarding COVID-19. All KAP scores were higher in male students. Significant difference of good practice (*p* < 0.001) was observed among students who were in high socioeconomic status (SES) group. In univariate analysis, female sex (β: -0.75, *p* = 0.007) was negatively and watching television (β: 0.44; *p* = 0.014) was positively associated with knowledge score. However, in multivariate linear regression analysis, the knowledge score was significantly associated with age (β: -0.115; *p* = 0.004); attitude score was significantly associated with female sex (β: -2.776; *p* < 0.001) and radio use (β: -0.974; *p* = 0.031); and practice score was significantly associated with female sex (β: -3.704; *p* = 0.002) and urban area (β: 1.702; *p* = 0.016).

**Conclusions:**

The overall knowledge and attitudes regarding COVID-19 among university students were desirable. Higher university year, young age, male sex and living in urban areas were significantly associated with good KAP regarding COVID-19. Good practices towards COVID-19 should be increased through awareness programs.

## Introduction

An outbreak of pneumonia-like cases with unknown cause appeared in December 2019 in the city of Wuhan, China and by January 7, 2020, scientists from China isolated the causative agent and introduced it as severe acute respiratory syndrome coronavirus 2 (SARS-CoV-2), also known as 2019-nCoV [1]. Later, World Health Organization (WHO) nominated this virus as coronavirus disease 2019 or simply COVID-19 [2]. It became evident that COVID-19 was transmitted from person to person and the clinical features were variant, ranging from being asymptomatic, moderate upper respiratory disease and to severe viral infection leading to respiratory failure and even death, hence causing an increased rate of hospitalization in Wuhan [3]. The COVID-19 is characterized by high morbidity and mortality rates and has caused the shut-down of social activities throughout the world which in turn has led a global economic fall [4].

Afghanistan, a war torn country with limited public health services, in particular limited COVID-19 diagnostic laboratories, and limited hospitals for in-patient services, is totally depended on preventive strategies such as complete/partial lockdown, using personal protective equipment and techniques to avoid or halt the spread of COVID-19 [5–7]. Furthermore, rapidly increasing cases of the COVID-19 which are officially confirmed by the ministry of public health and many self-reported cases due to the limited COVID-19 diagnostic services, precise numbers and figures are needed to avoid misconception and provide better work on public perception regarding the COVID-19 current situation in Afghanistan [8].

Public punctuality to accept and follow behavioral changes and adherence to preventive strategies are the key to mitigate and control the spread of the COVID-19. This could be achieved by increasing the level of knowledge, attitudes and practices (KAP) regarding COVID-19.

KAP studies about COVID-19 could be very helpful for evaluating the efficacy and success of public health education regarding the ongoing pandemic. In addition, appropriate knowledge, attitudes and practices toward COVID-19 among communities are of immense importance and are critical for the prevention of this pandemic. Hence, various studies have been conducted in many regions of the world, where the level of knowledge about COVID-19 is different among different countries [9]. Successful implementation of preventive measures and level of awareness in particular among university students in developing countries such as Afghanistan could play a crucial role in spreading the information regarding COVID-19 to the public for better tackling the ongoing pandemic [10, 11].

While the first confirmed case of COVID-19 was in a traveler from Iran in Herat province of Afghanistan on the 24^th^ of February, 2020, there were only a couple of diagnostic centers functioning in the beginning of the first wave of COVID19 in Afghanistan. A study conducted during the first wave of the pandemic in Kandahar, Afghanistan showed that due to low level of literacy, majority of the people considered COVID-19 as a rumor. Due to factors such as lack of health-related services and religious misbeliefs people think that death is already predetermined according to their destiny [12].

Relatively, similar conditions are reported in other low-income countries, such as Bangladesh, where factors of poor health infrastructure and services and insufficient knowledge have paved the way toward poor infection control that led to further spread of infection in the area [13, 14].

There is a strong need to tackle the above factors and increase the level of KAP regarding COVID19 particularly among university students in Afghanistan, who can play a crucial role in disseminating public health recommendations regarding mitigating COVID19 among their communities.

Few studies are published regarding COVID-19 in Afghanistan and to our knowledge, no KAP study has been published among university students. Therefore, the aim of this study was to determine the level of knowledge, attitudes, and practices regarding COVID-19 among Kandahar university students during the second wave of the COVID-19 pandemic.

## Material and methods

### Study design and setting

A cross-sectional study of knowledge, attitudes and practices of undergraduate students regarding COVID-19 during the second wave lockdown was conducted in Kandahar University (KDRU), a public higher education institute in Kandahar province, Afghanistan. The study was conducted from January to March, 2021 during the suspension of routine academic activities due to COVID-19 pandemic.

### Study participants

The study participants were all undergraduate students of KDRU who were under lockdown and were studying remotely from their homes. KDRU has around 8,500 students (91.3% male, 8.7% female) enrolled in 10 faculties, i.e., journalism, medicine, engineering, education, political sciences, Islamic studies, economics, language and literature, law, and computer science.

### Sample size calculation and sampling

Sample size calculation was based on the following formula: z2pq/d^2^ [15]. Using openEpi.com calculator, after considering the confidence level of 95%, with 5% margin of error, proportion of 50%, and the population size of 8,500 students, a total of 385 participants were estimated. However, a 10% non-response was added to reach the final sample size of 424.

For the recruitment of study participants, a simple random sampling method was used. For this purpose, initially a complete database of all students in KDRU was obtained and a simple random sample of 424 students was selected using statistical software Stata 14. Initially, contact numbers of the selected participants were obtained from their respected faculties, Then, each participant was contacted through mobile phone by the interviewer. If the participant refused to participate or did not reply the phone call, the subsequent student was selected from the database. At the end of survey, a total of 360 students were interviewed with a response rate of 84.9% and after excluding one participant with missing data, 359 participants were included in the final analysis.

### Data collection

As the universities were in lockdown, students were studying online. All study participants did not have sufficient access to internet facilities all the time due to war conflicts and economic problems. Therefore, the easy way to approach the study participants was contacting through mobile phones and social media groups (WhatsApp). Data were collected through phone call interviews using a structured questionnaire designed in Epi Info 7. Three medical doctors (2 males and 1 female) were trained for data collection prior to conducting the study.

### Data collection tool

The study questionnaire comprised of seven parts: (1) Socio-demographic characteristics; (2) Lifestyle history; (3) Physical activity level during last week; (4) Stress level using Patient Health Questionnaire (PHQ-9); (5) Personal medical background; (6) Knowledge, attitudes and practices (KAP) questionnaire regarding COVID-19; and (7) Effects of COVID-19 on students’ routine life. However, this questionnaire is part of a research project and we only utilized the sociodemographic and KAP related questionnaire tool for the current study.

The KAP questionnaire had 15 questions on knowledge about COVID-19, 10 questions on attitudes toward COVID-19, and 10 questions on practices relevant to COVID-19. The questionnaire was developed following an extensive literature review of the relevant studies [9, 11, 16–20]. Questions related to knowledge had three options (Yes, No, and I don’t know). One score was given for Yes and zero for No or I don’t know. Total knowledge score ranged from zero to 15 and was divided into 2 categories. An individual score of 0 to 11 was considered as poor/inadequate knowledge, and a score of 12 to 15 was taken as good/ adequate knowledge. Participants’ attitudes toward COVID-19 were assessed using a 5-point Likert scale. For each statement respondents were asked to state their level of agreement as strongly disagree, disagree, neutral, agree, strongly agree and the answers were awarded 1 to 5 scores. Total attitudes score ranged from 10 to 50 and an arbitrary cut-off of 70% was used to categorize the final attitudes score into less than 70% as a negative attitude and 70% or above as a positive attitude toward COVID-19. Practices toward COVID-19 were also calculated based on 5-point Likert scale and a score of 5 to 1 was assigned to each reply of always, often, sometimes, rarely, and never, respectively. Total practices score ranged from 10 to 50 and an arbitrary cut-off point of 70% was used. Participants with a total practice score of less than 70% were considered having poor practice, while a score of 70% or more was considered as positive practice.

### Data analysis

Statistical analyses were performed using Stata 14.1 (StataCorp LP, College Station, TX, USA). A *p*-value of less than 0.05 was considered statistically significant. Descriptive statistics were used to calculate frequencies and percentages, while Kruskal–Wallis test was used to compare the difference between means of different groups for selected KAP scores. Socioeconomic status was evaluated using family affluence score for adolescence and is described in details elsewhere [21]. Linear regression analysis was used to identify factors associated with KAP score. All items of KAP study were evaluated for internal reliability using Cronbach’s alpha. The coefficients for Cronbach’s alpha for knowledge, attitudes and practices toward COVID-19 were 0.607, 0.693 and 0.695, respectively.

## Results

### Socio-demographic characteristics

**Fig. 1:**
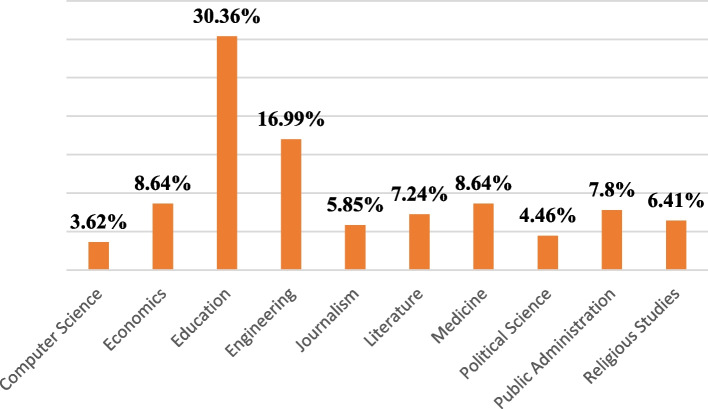
Percentage of study participants among different faculties of Kandahar University

From 359 participants, 88.9% were males, 81.3% were single, and 65.1% were living in urban areas. Almost two-thirds of the students were less than 23 years old. The general characteristics of the participants are shown in Table 1. Out of 10 faculties, one-third of the students were from Faculty of Education, while the lowest number of students (3.62%) were from the Faculty of Computer Science (Fig. 1). Taking parent’s education into account, 37% of the students’ fathers were uneducated compared to mothers among whom the illiteracy rate was around 80%.

**Table 1:** Sociodemographic Characteristics of the Study Participants (*N* = 359)

Variables	Frequency	Percentage
**Sex**		
Male	319	88.9
Female	40	11.1
**Residency**		
Rural	125	34.82
Urban	234	65.18
**Ethnicity**		
Pashtoon	293	81.62
Tajik	31	8.64
Hazara	15	4.18
Baloch	11	3.06
Sadat	7	1.95
Other	2	0.56
**Marital Status**		
Single	292	81.34
Married	67	18.66
**House Ownership**		
Private	296	82.45
Rent	53	14.76
Lease	5	1.39
Other	5	1.39
**Total Monthly Income (AFN)**		
< 10,000	68	18.94
10,000–20,000	179	49.86
20,000–30,000	70	19.5
> 30,000	42	11.7
**Father's Education**		
Uneducated	133	37.05
Elementary	63	17.55
High school	119	33.15
Religious	13	3.62
Higher education	31	8.64
**Mother's Education**		
Uneducated	293	81.62
Elementary	22	6.13
High school	22	6.13
Religious	19	5.29
Higher education	3	0.84
**Partner's Education**		
Uneducated	36	53.73
Elementary	16	23.88
High school	10	14.93
Religious	5	7.46
**Job**		
No	285	79.39
Yes	74	20.61
**Age Categories (years)**		
< 23	230	64.07
≥ 23	129	35.93
**Family Affluence Scale**		
Low SES	122	33.98
Moderate SES	170	47.35
High SES	67	18.66
**Source of Information Regarding COVID-19**		
Social Media	292	81.3
TV	220	61.3
Radio	210	58.5
Ministry of Public Health	105	29.3
Friends and Relatives	23	6.4
**Willingness to Vaccination**		
No	88	24.5
Yes	271	75.5
**PCR Confirmed COVID19 Cases**		
No	348	96.9
Yes	11	3.1
**Self-reported COVID19 Cases**		
No	303	84.4
Yes	56	15.6

Abbreviations: *AFN* Afghani, *COVID19* Coronavirus 2019, *SES* Socioeconomic status

Furthermore, 18.6% of the students were in the high socioeconomic status (SES), more than 80% had private houses and almost 20% of the students had part-time jobs. Regarding vaccination for COVID-19, 75.5% of the participants were willing to vaccine themselves if available. Self-reported prevalence of COVID-19 was 15.6%, however, PCR-confirmed cases reached only 3.1% of the study participants.

### Level of knowledge, attitudes, and practices toward COVID-19

#### Knowledge

Table 2 presents the knowledge of the students regarding COVID-19. Majority of the participants knew the causative agent of the COVID-19 (91.6%), person to person transmission distance (88.9%), spread through respiratory droplets (90.0%), and through touching contaminated objects (89.4%), the main clinical symptoms (91.6%), and that severe shortness of breath was considered as an immediate hospitalization sign (89.1%).

**Table 2:** Knowledge of the Study Participants Regarding COVID-19

	**Knowledge**	**Yes**	**No**	**I Don't Know**
		*N* (%)	*N* (%)	*N* (%)
K1	Causative organism of Corona Disease is a virus	329 (91.6)	24 (6.7)	6 (1.7)
K2	Corona virus spreads from person-to-person within close distance of each other (approx. six feet)	319 (88.9)	31 (8.6)	9 (2.5)
K3	Corona virus spread through respiratory droplets, which occur when infected people cough and sneeze	323 (90.0)	30 (8.3)	6 (1.7)
K4	Corona virus can be contracted by touching a contaminated object and then touching one’s mouth or nose	321 (89.4)	28 (7.8)	10 (2.8)
K5	Is it possible that someone gets Corona Disease from asymptomatic person?	220 (61.3)	89 (24.8)	50 (13.9)
K6	The main clinical symptoms of Corona Disease are fever, chills, fatigue, dry cough, myalgia and shortness of breath	327 (91.1)	23 (6.4)	9 (2.5)
K7	Severe shortness of breath is a warning sign for going to the hospital	320 (89.1)	26 (7.3)	13 (3.6)
K8	Antibiotics are an effective treatment for Corona Disease	209 (58.2)	41 (11.4)	109 (30.4)
K9	Older people and those with heart or lung disease and diabetes, are at increased risk	314 (87.5)	27 (7.5)	18 (5.0)
K10	Children do not appear to be at higher risk for Corona Disease than adults	293 (81.6)	50 (13.9)	16 (4.5)
K11	Washing hands with soap for at least 20 s can help in COVID-19 prevention	338 (94.2)	16 (4.4)	5 (1.4)
K12	Wearing masks in public places can prevent the Corona virus infection	331 (92.2)	25 (7.0)	3 (0.8)
K13	Healthy food and fruits increase the body’s immunity to Corona Disease	331 (92.2)	24 (6.7)	4 (1.1)
K14	Isolation of people infected with COVID-19 is effective way to reduce the spread of virus	328 (91.4)	26 (7.2)	5 (1.4)
K15	People in contact with someone infected with COVID-19 should be quarantined for 14 days	298 (83.0)	27 (7.5)	34 (9.5)

In addition, 87.5% of the participants knew that older people and those who suffer from chronic diseases are at higher risk of getting the COVID-19. Participants also knew that washing hands for 20 s regularly (94.2%), wearing face masks in public places (92.2), isolation from infected persons (91.4%), eating healthy food (92.2), and quarantine for 14 days (83.0) are important protective measurements advised by public health authorities. More than half of the participants (58.2%) thought that antibiotics are effective treatment for COVID-19 and a quarter (24.8%) of them did not think that COVID-19 could be contracted from asymptomatic persons.

#### Attitudes

Table 3 summarizes the attitude of the participants towards COVID-19. Majority of the students agreed to keep distance from others, wash hands and not visit crowded places, and that COVID-19 was a true and deadly disease and seeking help was important for suspected cases. Moderately positive attitude was noted among the students regarding successful control of the COVID-19. Less than 60% of the study participants agreed that the government services were satisfactory for controlling the COVID-19.

**Table 3:** Attitudes of the Study Participants Regarding COVID-19

	**Attitude**	**Strongly Disagree**	**Disagree**	**Neutral**	**Agree**	**Strongly agree**
A1	It is important to keep my distance from others, to avoid spreading Corona Disease	7 (1.9)	15 (4.2)	3 (0.8)	183 (51.0)	151 (42.1)
A2	Washing hands is essential to protect myself from Corona Disease	3 (0.8)	2 (0.6)	5 (1.4)	246 (68.5)	103 (28.7)
A3	To protect myself from COVID-19, I should not visit crowded places	1 (0.3)	12 (3.3)	10 (2.8)	208 (57.9)	128 (35.7)
A4	COVID-19 will eventually be successfully controlled	4 (1.1)	9 (2.5)	72 (20.1)	223 (62.1)	51 (14.2)
A5	Strict measures (lockdown) can help win the battle against COVID-19	22 (6.1)	57 (15.9)	9 (2.5)	166 (46.2)	105 (29.3)
A6	Your family members are concerned about getting Corona Disease?	43 (12.0)	63 (17.5)	53 (14.8)	168 (46.8)	32 (8.9)
A7	Corona Disease is a dangerous and deadly disease	5 (1.4)	11 (3.1)	11 (3.1)	170 (47.3)	162 (45.1)
A8	It is important to seek immediate medical care, if you are suspected to have Corona Disease	2 (0.6)	17 (4.7)	11 (3.1)	193 (53.7)	136 (37.9)
A9	Corona Disease is a true disease	0 (0)	5 (1.4)	19 (5.3)	265 (73.8)	70 (19.0)
A10	Government’s overall services regarding Corona Disease are satisfactory	11 (3.1)	45 (12.5)	89 (24.8)	191 (53.2)	23 (6.4)

One-third of the study participant’s family members were not concerned about getting COVID-19. When students asked if lockdown could be helpful in winning the battle against COVID-19, 22% showed disagreement, while 75.2% were in favor of lockdown to tackle the COVID-19.

##### Practices

The students’ practices towards COVID-19 are described in Table 4. Almost half of the participants washed or disinfected their hands always and around one-third of them shook hands or hugged while greeting people. One-fifth of the participants always avoided touching faces and eyes, used face masks, followed the news about COVID-19 and avoided social gatherings. Almost 10% of the participants disinfected surfaces and objects, and kept 1–1.5 m of distance almost all the time. Furthermore, shaking hands and hugging practices when greeting others were avoided by only 9.2% and 10.6% of the students, respectively. Moreover, almost one-third of the students never kept the recommended distance of 1 to 1.5 m from others and 12.8% reported that they shared the essential information regarding COVID-19 to public regularly while 19.2% of the students did this only sometimes. Only 6.7% of the students avoided social gatherings.

**Table 4:** Practices of the Study Participants Regarding COVID-19

	**Practice**	**Always**	**Often**	**Sometimes**	**Rarely**	**Never**
P1	How often do you wash or disinfect your hands?	160 (44.6)	101 (28.1)	82 (22.9)	12 (3.3)	4 (1.1)
P2	How often do you avoid touching your face and eyes with unwashed hands?	81 (22.6)	101 (28.1)	133 (37.0)	34 (9.5)	10 (2.8)
P3	How often do you use a face mask while visiting crowded places?	75 (20.8)	85 (23.7)	132 (36.8)	28 (7.8)	39 (10.9)
P4	How often do you regularly disinfect surfaces of personal objects and places?	36 (10.0)	74 (20.6)	165 (46.0)	31 (8.6)	53 (14.8)
P5	How often do you shake hands with others?	108 (30.1)	82 (22.8)	85 (23.7)	51 (14.2)	33 (9.2)
P6	How often do you hug others?	107 (29.8)	39 (10.8)	113 (31.5)	62 (17.3)	38 (10.6)
P7	How often do you keep at least 1–1.5 m distance from others?	30 (8.4)	58 (16.1)	107 (29.8)	60 (16.7)	104 (29.0)
P8	How often do you follow the news about Corona Disease?	80 (22.3)	75 (20.9)	148 (41.2)	28 (7.8)	28 (7.8)
P9	How often do you try to teach other people about the prevention of getting Corona Disease?	46 (12.8)	69 (19.2)	157 (43.8)	54 (15.0)	33 (9.2)
P10	How often do you avoid social gatherings?	67 (18.7)	70 (19.5)	134 (37.3)	64 (17.8)	24 (6.7)

**Fig. 2:**
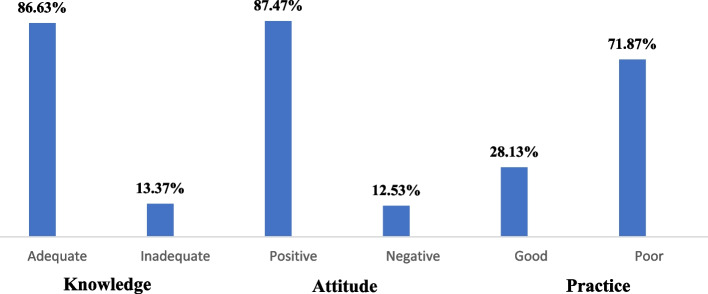
Knowledge, attitudes and practices of Kandahar University students towards COVID-19

Overall, 86.6% of the study participants had adequate level of knowledge regarding COVID-19 and 87.5% of them had positive attitudes toward COVID-19. Further, almost one-third (28.2%) of the participants had good practices regarding COVID-19, whereas 71.8% reported poor practices against the COVID-19 (Fig. 2).

##### Comparison of sociodemographic characteristics with mean KAP scores

The associations between sociodemographic characteristics and mean KAP scores are summarized in Table 5. In this study, the overall score for Knowledge was 12.8 ± 1.6 (range: 0 ~ 15), Attitudes score was 39.8 ± 3.8 (range: 10 ~ 50), and Practices score was 31.6 ± 6.0 (range: 10 ~ 50). A significant difference of Knowledge (*p* < 0.001), Attitudes (*p* < 0.001) and Practices (*p* = 0.007) was noted between male and female students. All KAP scores were higher in male students as compared to female students. A significant difference of good practice (*p* < 0.001) was observed among students who were in high socioeconomic status (SES) group, while no statistically significant difference was seen in knowledge and attitude regarding COVID-19. The overall Attitude and Practice scores were significantly different (*p* < 0.001) among students of different faculties, while the difference of Knowledge score was borderline (*p* < 0.056). Students of Medicine Faculty had the highest attitude score (43.2 ± 3.7), while students of Computer Science Faculty had the lowest (35.2 ± 4.5). However, significantly good practice (35.0 ± 4.9) was observed in the students of Faculty of Literature. A significant result was identified in the case of attitude score category across the university years, where fifth year students were found to have the most positive attitude (42.9 ± 4.2, *p* < 0.001) compared to the rest of students, while knowledge and practices were not significantly different among the university years. No significant differences were observed among KAP scores for age, marital status, and living areas.

**Table 5:** Comparison of sociodemographic characteristics and mean KAP score

**Variable**	**N**	**%**	**Knowledge**			**Attitudes**			**Practices**		
			Mean	SD	*P* ^a^	Mean	SD	*P* ^a^	Mean	SD	*P* ^a^
**Gender**											
Female	40	11.1	12.2	1.0	** < 0.001**	37.9	2.4	** < 0.001**	29.4	2.4	**0.007**
Male	319	88.9	12.9	1.7		40.0	3.9		31.9	6.3	
Total	359	100	12.8	1.6		39.8	3.8		31.6	6.0	
**Age**											
< 23	230	64.1	12.8	1.7	0.429	39.9	3.8	0.219	31.8	6.3	0.427
≥ 23	129	35.9	12.9	1.6		39.5	4.0		31.3	5.5	
Total	359	100	12.8	1.6		39.8	3.8		31.6	6.0	
**Marital Status**											
Single	292	81.3	12.8	1.7	0.879	39.6	3.7	0.122	31.5	6.1	0.154
Married	67	18.7	12.8	1.6		40.3	4.3		32.4	5.6	
Total	359	100	12.8	1.6		39.8	3.8		31.6	6.0	
**Socioeconomic Status (SES)**											
Low SES	122	34.0	12.7	2.0	0.890	39.7	3.6	0.058	32.6	6.2	** < 0.001**
Moderate SES	170	47.4	12.9	1.3		39.4	4.2		30.4	5.6	
High SES	67	18.7	12.8	1.6		40.7	3.1		33.0	6.3	
Total	359	100	12.8	1.6		39.8	3.8		31.6	6.0	
**Living Area**											
Rural	125	34.8	12.9	2.0	0.069	39.7	3.9	0.957	30.9	6.1	0.133
Urban	234	65.2	12.8	1.4		39.8	3.8		32.0	6.0	
Total	359	100	12.8	1.6		39.8	3.8		31.6	6.0	
**Faculty**											
Computer Science	13	3.6	12.5	1.1	< 0.059	35.2	4.5	< 0.001	27.6	6.3	< 0.001
Economics	31	8.6	13.1	1.1		37.4	2.6		26.7	4.0	
Education	109	30.4	12.8	1.5		40.7	2.9		34.7	5.6	
Engineering	61	17.0	13.0	1.1		39.5	3.2		28.2	4.0	
Journalism	21	5.9	13.0	1.6		41.8	2.7		34.2	6.7	
Literature	26	7.2	12.6	2.5		41.3	3.2		35.0	4.9	
Medicine	31	8.6	13.2	1.9		43.2	3.7		32.8	5.4	
Political Science	16	4.5	13.5	1.3		35.6	2.4		27.8	4.0	
Public Administration	28	7.8	12.4	0.9		35.9	3.0		28.1	3.8	
Religious Studies	23	6.4	11.9	2.8		40.9	4.2		34.2	6.2	
Total	359	100	12.8	1.6		39.8	3.8		31.6	6.0	
**Faculty Year**											
1	107	29.8	12.6	2.0	0.385	39.7	3.8	< 0.001	32.2	6.5	0.330
2	79	22.0	12.8	1.4		39.6	3.1		31.9	6.1	
3	72	20.1	13.1	1.3		40.7	3.9		31.8	5.9	
4	88	24.5	12.8	1.5		38.7	4.1		30.5	5.5	
5	13	3.6	12.9	2.5		42.9	4.2		32.0	6.3	
Total	359	100	12.8	1.6		39.8	3.8		31.6	6.0	

Notes: ^a^Kruskal-Wallis test

Abbreviations: KAP, knowledge, attitudes and practices; SES, socioeconomic status

##### Factors associated with KAP scores

Linear regression analysis was performed to investigate the relationship of associated factors with KAP scores (Table 6). The univariate analysis showed that female sex (β: -0.75, *p* = 0.007) and watching television (β: 0.44; *p* = 0.014) were significantly associated with knowledge score. While female sex (β: -2.091; *p* = 0.001) and information through Ministry of Public Health (β: 0.854; *p* = 0.055) were significantly associated with attitude score, only female sex (β: -2.568; *p* = 0.011) was associated with practice scores.

**Table 6:** Linear regression analysis results of KAP related factors for COVID-19

Unadjusted Regression Analysis												
**Variable**	**Knowledge**				**Attitudes**				**Practices**			
	**Coef**	**95% CI**		***P***	**Coef**	**95% CI**		***P***	**Coef**	**95% CI**		***P***
**Female**	-0.750	-1.288	-0.211	**0.007**	-2.091	-3.339	-0.842	**0.001**	-2.568	-4.548	-0.589	**0.011**
**Age**	-0.051	-0.114	0.013	0.117	-0.042	-0.190	0.106	0.576	-0.074	-0.307	0.159	0.535
**Marital Status**	-0.031	-0.470	0.409	0.89	0.647	-0.375	1.668	0.214	0.966	-0.645	2.576	0.239
**Socioeconomic Status**	0.023	-0.086	0.132	0.676	0.067	-0.187	0.321	0.604	0.032	-0.369	0.432	0.876
**Urban Area**	-0.110	-0.470	0.249	0.547	0.033	-0.804	0.870	0.938	1.117	-0.197	2.432	0.095
**Faculty Year**	0.063	-0.074	0.200	0.368	0.035	-0.285	0.355	0.829	-0.417	-0.920	0.085	0.103
**Social Media**	0.398	-0.040	0.835	0.075	0.509	-0.513	1.532	0.328	0.007	-1.607	1.620	0.993
**Television**	0.440	0.091	0.788	**0.014**	0.168	-0.651	0.987	0.687	0.609	-0.680	1.898	0.353
**Radio**	0.328	-0.018	0.674	0.063	-0.322	-1.131	0.486	0.433	0.243	-1.032	1.519	0.708
**Ministry of Public Health**	0.273	-0.102	0.649	0.153	0.854	-0.018	1.726	**0.055**	-0.369	-1.750	1.013	0.600
**Friends and Relatives**	-0.268	-0.967	0.431	0.451	0.909	-0.717	2.535	0.272	1.136	-1.428	3.701	0.384
**Adjusted Multivariate Regression Analysis**												
**Variable**	**Knowledge**				**Attitude**				**Practice**			
	**Coef**	**95% CI**		***P***	**Coef**	**95% CI**		***P***	**Coef**	**95% CI**		***P***
**Female**	-0.583	-1.223	0.058	0.075	-2.776	-4.269	-1.283	** < 0.001**	-3.704	-6.068	-1.341	**0.002**
**Age**	-0.115	-0.194	-0.037	**0.004**	-0.169	-0.352	0.013	0.069	-0.073	-0.361	0.216	0.621
**Marital Status**	0.137	-0.346	0.620	0.577	0.817	-0.308	1.942	0.154	1.024	-0.757	2.805	0.259
**Socioeconomic Status**	-0.010	-0.127	0.107	0.864	-0.025	-0.298	0.248	0.855	-0.012	-0.444	0.420	0.957
**Urban Area**	-0.068	-0.445	0.308	0.722	0.303	-0.575	1.181	0.498	1.702	0.313	3.091	**0.016**
**Faculty Year**	0.151	-0.005	0.307	**0.058**	0.208	-0.156	0.572	0.262	-0.360	-0.936	0.217	0.220
**Social Media**	0.194	-0.279	0.666	0.421	-0.295	-1.397	0.807	0.599	-0.614	-2.358	1.130	0.489
**Television**	0.310	-0.060	0.680	0.101	-0.137	-1.000	0.725	0.754	0.466	-0.900	1.831	0.503
**Radio**	0.185	-0.194	0.565	0.337	-0.974	-1.860	-0.089	**0.031**	-0.307	-1.708	1.095	0.667
**Ministry of Public Health**	0.157	-0.240	0.554	0.437	0.692	-0.234	1.617	0.143	-0.971	-2.435	0.494	0.193
**Friends and Relatives**	-0.413	-1.118	0.292	0.250	0.722	-0.922	2.366	0.388	1.299	-1.302	3.901	0.327

In multivariate linear regression analysis, the knowledge score was significantly associated with age (β: -0.115; *p* = 0.004); attitudes score was significantly associated with female sex (β: -2.776; *p* < 0.001) and radio use (β: -0.974; *p* = 0.031); and practices score was significantly associated with female sex (β: -3.704; *p* = 0.002) and urban area (β: 1.702; *p* = 0.016).

## Discussion

This study was conducted during the second wave of the COVID-19 pandemic in Afghanistan to assess the KAP of Kandahar university students regarding COVID-19.

Majority of the study participants (91.6%) knew that the causative agent of the ongoing pandemic was a virus which is similar finding reported in a study conducted in Bangladesh [13]. However, our finding is higher than what is reported from Pakistan [11]. The difference could be due to the second wave of the pandemic in Afghanistan. Nonetheless, in a study in the United Arab Emirates (UAE), slightly higher proportion of the students (95.6%) were familiar with the causative agent [22].

Almost more than half (58.2%) of the students were in favor of using antibiotics for treatment of the COVID-19. A higher number of university students (66.2%) from India has the same misinformation. This is a worrying finding which could lead to irrational use of antibiotics in the country and may contribute to antibiotic resistance [23]. However, in a study in the UAE, 13.2% of students were in favor of using antibiotics for COVID-19 patients [22]. This difference may be due to the huge number of non-medical participants in our study and overall disbelief on the treatment and management of COVID-19 in the region.

Social media was the main source of information for more than 81% of the students as compared to other sources of information such as television, MoPH sources, etc. This is in line with a study conducted in the UAE [22]. This could be explained by the fact that university students are more familiar with the social media as an easy and important way of access to knowledge.

Majority (87.4%) of the participants in our study had positive attitudes toward the protective measurements against COVID-19, which is higher (61.4%) compared to a study conducted among university students in Bangladesh [13]. In addition, the level of positive attitude is less than a study conducted among medical students in Pakistan [11] and India [24]. However, the report is higher than what is reported from community pharmacists in Pakistan [25], and the public in Nigeria (79.5%) [4], and Iran (50%) [16].

In addition, the knowledge, attitude and practice scores were higher in males than in females. These findings were similar to a study conducted among medical students in Malaysia [26]. Regarding faculty years, though students in higher years of studies had higher knowledge, attitudes and practices scores than students in lower years, only attitude score was significantly associated with faculty years. These findings were also in line with other studies in Uganda and Malaysia [26, 27].

Overall, good practices were reported by 28.2% of the university students, while 72.2% experienced high risk or poor practices. A slightly higher findings (34.4%) of good practices were observed in a study among university students in Bangladesh [28]. The level of appropriate practices among Kandahar university students is quite low compared to what is reported on medical students in India (90%), Pakistan (95%), and China (87.9%) [11, 29, 30], and among pharmacy workers in Pakistan (57.5%) [25]. The high level of poor practices among university students is alarming, as they can mislead other people by neglecting preventive practices. This in turn may cause further transmission of the disease.

In our study, among KAP related factors age was significantly associated with knowledge score, which is similar to studies conducted elsewhere [9, 29, 31, 32]. In Afghanistan, younger students are more adapted to modern technologies compared to older students. Hence, they may have more adequate information about COVID-19. In addition, in our study the levels of attitudes and practices were better in female students than male students regarding COVID-19. Similar findings were reported in different studies among university students in Pakistan, Korea, China and Japan [11, 19, 33]. This could be described by studies, which have reported significant differences between male and female participants regarding protective public health measures in context to infectious diseases. Such studies suggest that the level of preventative measures of female adults tends to be higher than that of males [34, 35].

Self-reported prevalence of the COVID-19 was 15.6%, higher than PCR-confirmed cases (3.1%). This could be explained by the fact that beside other challenges, few reliable COVID-19 diagnostic centers are available throughout the country. Considering the country’s population and the ongoing COVID-19 emergency, the testing capacity could not meet the needs, hence, testing practice among the study participants was reported very low [6, 7, 36].

The willingness to get the COVID-19 vaccine was 75.5% among the participants. This rate is higher than the studies in Japan, UK and the UAE [37–39].

A community-based KAP study regarding COVID-19 in Kandahar city found that the awareness and practices of the people towards the COVID-19 were significantly low. Despite local public health partial preventive measures in Kandahar city, initial response in the community to the threat of COVID-19 was unconcerned, such as social distancing was completely violated in the ceremonies like wedding gathering, in prayer times in the mosques, and even in reopening of the universities and schools. According to the author, majority of the people considered COVID-19 as rumor due to lack of sufficient knowledge as well as religious beliefs. This may have contributed to poor practices regarding COVID-19 among public in Kandahar [12].

This study had some limitations. First, the total number of female students were less than male in the university. Second, the nature of cross-sectional design is unable to determine causality between the variables. Third, the participants were enrolled only from one public university in Kandahar, therefore, the findings might not reflect the actual situation of undergraduate university students in Kandahar and in Afghanistan at large. Finally, the study was conducted during the second wave of the COVID-19 in Afghanistan, which may have affected the knowledge, attitude and practice level of the study participants.

## Conclusion

The overall knowledge and attitudes of university students regarding COVID-19 are appropriate. Higher university year, young age, male sex and living in urban areas are the factors associated with good KAP regarding COVID-19. However, majority of the students had poor practices regarding COVID-19 which is alarming. The university administration and other stake holders should work closely to change the students' mindsets and convince them to abide by preventive practices to make sure their negligence does not affect themselves and most importantly the high-risk groups in their families and the society at large.

## Data Availability

Data are available on reasonable request from the corresponding author.

## Abbreviations

COVID-19: Coronavirus disease 2019
IRB: Institutional review board
KAP: Knowledge, attitudes and practices
SES: Socioeconomic status
WHO: World Health Organization
MOPH: Ministry of public health
AFN: Afghani
